# 1-(4-Chloro­phen­yl)-3-(2-thienylcarbon­yl)thio­urea

**DOI:** 10.1107/S1600536810013863

**Published:** 2010-04-24

**Authors:** Sohail Saeed, Naghmana Rashid, Wing-Tak Wong

**Affiliations:** a Department of Chemistry, Research Complex, Allama Iqbal Open University, Islamabad, Pakistan; b Department of Applied Biology and Chemical Technology, The Hong Kong Polytechnic University, Hung Hom, Kowloon, Hong Kong SAR, People’s Republic of China

## Abstract

The title compound, C_12_H_9_ClN_2_OS_2_, exists in the thio­amide form with an intra­molecular N—H⋯O hydrogen bond across the thio­urea and the carbonyl group. The dihedral angle between the rings is 10.36 (11)°. In the crystal structure, mol­ecules are linked into chains by weak inter­molecular C—H⋯Cl hydrogen-bonding inter­actions.

## Related literature

For general background to the biological activity of thio­urea derivatives, see: Xu *et al.* (2004 ▶); Gu *et al.* (2007 ▶). For related structures, see: Saeed *et al.* (2008 ▶, 2009 ▶). For the cytotoxicity of anti­cancer drugs to normal cells in cancer therapy, see: Saeed *et al.* (2010 ▶).
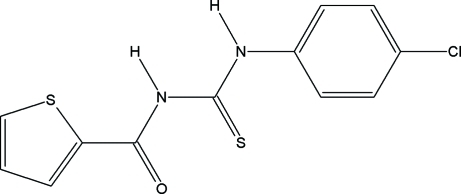


## Experimental

### 

#### Crystal data

C_12_H_9_ClN_2_OS_2_
*M* *_r_* = 296.78
Monoclinic, 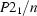

*a* = 4.6552 (7) Å
*b* = 11.660 (2) Å
*c* = 23.630 (4) Å
β = 95.626 (2)°
*V* = 1276.4 (4) Å^3^
*Z* = 4
Mo *K*α radiation
μ = 0.61 mm^−1^
*T* = 300 K
0.42 × 0.19 × 0.08 mm

#### Data collection

Bruker SMART 1000 CCD diffractometer
Absorption correction: multi-scan (*SADABS*; Sheldrick, 1996 ▶) *T*_min_ = 0.783, *T*_max_ = 0.953
8549 measured reflections
3102 independent reflections
2578 reflections with *I* > 2σ(*I*)
*R*_int_ = 0.019

#### Refinement

*R*[*F* ^2^ > 2σ(*F* ^2^)] = 0.038
*wR*(*F* ^2^) = 0.107
*S* = 1.07
3102 reflections
172 parameters
H atoms treated by a mixture of independent and constrained refinement
Δρ_max_ = 0.31 e Å^−3^
Δρ_min_ = −0.20 e Å^−3^

### 

Data collection: *SMART* (Bruker, 1998 ▶); cell refinement: *SAINT* (Bruker, 2006 ▶); data reduction: *SAINT* and *CrystalStructure* (Rigaku/MSC and Rigaku, 2006 ▶); program(s) used to solve structure: *SHELXS97* (Sheldrick, 2008 ▶); program(s) used to refine structure: *SHELXL97* (Sheldrick, 2008 ▶); molecular graphics: *ORTEPII* (Johnson, 1976 ▶) and *DIAMOND* (Brandenburg, 1998 ▶); software used to prepare material for publication: *SHELXL97*.

## Supplementary Material

Crystal structure: contains datablocks global, I. DOI: 10.1107/S1600536810013863/lx2142sup1.cif

Structure factors: contains datablocks I. DOI: 10.1107/S1600536810013863/lx2142Isup2.hkl

Additional supplementary materials:  crystallographic information; 3D view; checkCIF report

## Figures and Tables

**Table 1:** Hydrogen-bond geometry (Å, °)

*D*—H⋯*A*	*D*—H	H⋯*A*	*D*⋯*A*	*D*—H⋯*A*
N1—H1*N*⋯O1	0.87 (2)	1.91 (2)	2.651 (2)	143 (2)
C12—H12⋯Cl1^i^	0.93	2.69	3.523 (2)	149

Symmetry code: (i) 
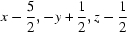
.

**Fig. 1.**
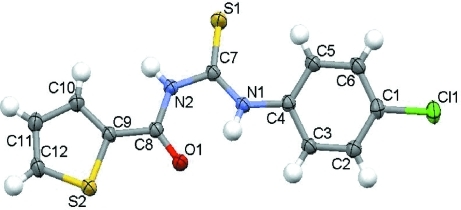
The molecular structure of the title compound with the atom numbering scheme. Displacement ellipsoids are drawn at the 30% probability level. H atoms are presented as a small spheres of arbitrary radius.

**Fig. 2.**
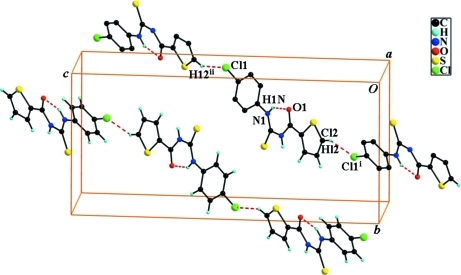
N–H···O and C–H···Cl interactions (dotted lines) in the crystal structure of the title compound. [Symmetry codes: (i) x - 5/2, - y + 1/2, z - 1/2 (ii) x + 5/2, - y + 1/2, z + 1/2.]

## supplementary crystallographic information

### Comment

Thiourea and its derivatives are an important class of organic compounds in which sulfur is the major ligand atom which plays an important role in coordination chemistry with transition metals. Thiourea and its derivatives have found extensive applications in the fields of medicine, agriculture and analytical chemistry. Thioureas are also known to exhibit a wide range of biological activities including anticancer (Saeed *et al.*, 2010), antifungal (Saeed *et al.*, 2008), antiviral, antibacterial, anti-tubercular, anti-thyroidal, herbicidal and insecticidal activities, organocatalyst (Gu *et al.*, 2007) and as agrochemicals (Xu *et al.*, 2004).

The 4-chlorophenyl ring is slightly twisted {15.04 (8)°} from the thiourea plane. The thioureido group is also slightly twisted {5.0 (1)°} from the thiophene ring plane of S2/C9/C10/C11/C12. The molecular packing (Fig. 2) exhibits the thioamide form with an intramolecular N–H···O hydrogen bond across the thiourea system, with a N1–H1N···O1 (Table 1). The crystal packing (Fig. 2) is stabilized by weak intermolecular C–H···Cl hydrogen bonds between the thiophene H atom and the chlorine of an adjacent molecule, with a C12–H12···Cl1^i^ (Table 1).

### Experimental

A solution of 2-thiophenecarbonyl chloride (0.01 mol) in anhydrous acetone (80 ml) was added dropwise to a suspension of ammonium thiocyanate (0.01 mol) in anhydrous acetone (50 ml) and the reaction mixture was refluxed for 50 minutes. After cooling to room temperature, a solution of 4-chloroaniline (0.01 mol) in dry acetone (25 ml) was added and the resulting mixture refluxed for 2 h. The reaction mixture was poured into five times its volume of cold water, upon which the thiourea precipitated. The product was recrystallized from ethanol as white block crystals.

### Refinement

The H atoms bound C atoms were located from difference Fourier map and refined freely. All H atoms of C atoms were positioned geometrically and refined using a riding model, with C–H = 0.93 Å for aryl and thiophenyl H atoms. *U*_iso_(H) = 1.2*U*_eq_(C) for aryl thiophenyl H atoms.

### Crystal data

**Table:**

C_12_H_9_ClN_2_OS_2_	*F*(000) = 608
*M**_r_* = 296.78	*D*_x_ = 1.544 Mg m^−^^3^
Monoclinic, *P*2_1_/*n*	Melting point: 412 K
Hall symbol: -P 2yn	Mo *K*α radiation, λ = 0.71073 Å
*a* = 4.6552 (7) Å	Cell parameters from 8801 reflections
*b* = 11.660 (2) Å	θ = 1.7–28.3°
*c* = 23.630 (4) Å	µ = 0.61 mm^−^^1^
β = 95.626 (2)°	*T* = 300 K
*V* = 1276.4 (4) Å^3^	Prism, yellow
*Z* = 4	0.42 × 0.19 × 0.08 mm

### Data collection

**Table:**

Bruker SMART 1000 CCD diffractometer	3102 independent reflections
Radiation source: fine-focus sealed tube	2578 reflections with *I* > 2σ(*I*)
graphite	*R*_int_ = 0.019
ω scan	θ_max_ = 28.3°, θ_min_ = 1.7°
Absorption correction: multi-scan (*SADABS*; Sheldrick, 1996)	*h* = −6→6
*T*_min_ = 0.783, *T*_max_ = 0.953	*k* = −9→15
8549 measured reflections	*l* = −30→31

### Refinement

**Table:**

Refinement on *F*^2^	Primary atom site location: structure-invariant direct methods
Least-squares matrix: full	Secondary atom site location: difference Fourier map
*R*[*F*^2^ > 2σ(*F*^2^)] = 0.038	Hydrogen site location: inferred from neighbouring sites
*wR*(*F*^2^) = 0.107	H atoms treated by a mixture of independent and constrained refinement
*S* = 1.07	*w* = 1/[σ^2^(*F*_o_^2^) + (0.0546*P*)^2^ + 0.3548*P*] where *P* = (*F*_o_^2^ + 2*F*_c_^2^)/3
3102 reflections	(Δ/σ)_max_ < 0.001
172 parameters	Δρ_max_ = 0.31 e Å^−^^3^
0 restraints	Δρ_min_ = −0.20 e Å^−^^3^

### Special details

**Table:**

Geometry. All esds (except the esd in the dihedral angle between two l.s. planes) are estimated using the full covariance matrix. The cell esds are taken into account individually in the estimation of esds in distances, angles and torsion angles; correlations between esds in cell parameters are only used when they are defined by crystal symmetry. An approximate (isotropic) treatment of cell esds is used for estimating esds involving l.s. planes.
Refinement. Refinement of F^2^ against ALL reflections. The weighted R-factor wR and goodness of fit S are based on F^2^, conventional R-factors R are based on F, with F set to zero for negative F^2^. The threshold expression of F^2^ > 2sigma(F^2^) is used only for calculating R-factors(gt) etc. and is not relevant to the choice of reflections for refinement. R-factors based on F^2^ are statistically about twice as large as those based on F, and R- factors based on ALL data will be even larger.

### Fractional atomic coordinates and isotropic or equivalent isotropic displacement parameters (Å^2^)

**Table:**

	*x*	*y*	*z*	*U*_iso_*/*U*_eq_	
Cl1	1.37357 (11)	0.14501 (5)	0.54454 (2)	0.06174 (17)	
S1	0.42417 (13)	0.54355 (4)	0.38816 (3)	0.06558 (19)	
S2	−0.38487 (12)	0.23708 (4)	0.20193 (2)	0.05870 (17)	
O1	0.0745 (3)	0.22204 (11)	0.29637 (6)	0.0549 (3)	
N1	0.4375 (3)	0.31315 (13)	0.37757 (6)	0.0426 (3)	
H1N	0.357 (5)	0.2572 (18)	0.3578 (9)	0.053 (6)*	
N2	0.0880 (3)	0.41119 (13)	0.32158 (7)	0.0446 (3)	
H2N	0.001 (5)	0.475 (2)	0.3134 (10)	0.063 (7)*	
C1	1.1034 (4)	0.19841 (16)	0.49542 (8)	0.0448 (4)	
C2	0.9940 (4)	0.12913 (16)	0.45099 (8)	0.0470 (4)	
H2	1.0655	0.0554	0.4470	0.056*	
C3	0.7761 (4)	0.17123 (15)	0.41242 (7)	0.0446 (4)	
H3	0.7010	0.1254	0.3823	0.053*	
C4	0.6685 (4)	0.28161 (15)	0.41829 (7)	0.0395 (3)	
C5	0.7819 (4)	0.34955 (16)	0.46329 (8)	0.0486 (4)	
H5	0.7111	0.4233	0.4677	0.058*	
C6	1.0007 (4)	0.30746 (17)	0.50170 (8)	0.0506 (4)	
H6	1.0777	0.3531	0.5317	0.061*	
C7	0.3216 (4)	0.41540 (14)	0.36378 (7)	0.0409 (4)	
C8	−0.0182 (4)	0.32019 (15)	0.28912 (7)	0.0408 (4)	
C9	−0.2510 (4)	0.34711 (15)	0.24497 (7)	0.0413 (4)	
C10	−0.3865 (4)	0.44880 (17)	0.22982 (8)	0.0499 (4)	
H10	−0.3439	0.5183	0.2481	0.060*	
C11	−0.5988 (5)	0.43535 (18)	0.18316 (9)	0.0574 (5)	
H11	−0.7109	0.4952	0.1671	0.069*	
C12	−0.6202 (5)	0.32616 (19)	0.16464 (9)	0.0586 (5)	
H12	−0.7503	0.3022	0.1346	0.070*	

### Atomic displacement parameters (Å^2^)

**Table:**

	*U*^11^	*U*^22^	*U*^33^	*U*^12^	*U*^13^	*U*^23^
Cl1	0.0533 (3)	0.0688 (3)	0.0585 (3)	−0.0017 (2)	−0.0177 (2)	0.0113 (2)
S1	0.0745 (4)	0.0364 (2)	0.0783 (4)	−0.0039 (2)	−0.0311 (3)	−0.0049 (2)
S2	0.0706 (3)	0.0434 (3)	0.0563 (3)	0.0030 (2)	−0.0228 (2)	−0.0074 (2)
O1	0.0672 (9)	0.0381 (6)	0.0547 (8)	0.0023 (6)	−0.0187 (6)	−0.0028 (6)
N1	0.0463 (8)	0.0358 (7)	0.0429 (8)	−0.0042 (6)	−0.0088 (6)	−0.0016 (6)
N2	0.0469 (8)	0.0372 (7)	0.0466 (8)	0.0002 (6)	−0.0105 (6)	−0.0020 (6)
C1	0.0384 (8)	0.0518 (10)	0.0427 (9)	−0.0060 (7)	−0.0035 (7)	0.0076 (7)
C2	0.0494 (10)	0.0429 (9)	0.0473 (9)	0.0012 (8)	−0.0023 (7)	0.0025 (7)
C3	0.0491 (9)	0.0403 (9)	0.0423 (9)	−0.0046 (7)	−0.0060 (7)	−0.0026 (7)
C4	0.0387 (8)	0.0392 (8)	0.0394 (8)	−0.0047 (6)	−0.0026 (6)	0.0029 (7)
C5	0.0552 (10)	0.0416 (9)	0.0465 (9)	−0.0005 (8)	−0.0075 (8)	−0.0035 (7)
C6	0.0526 (10)	0.0501 (10)	0.0460 (9)	−0.0084 (8)	−0.0113 (8)	−0.0045 (8)
C7	0.0418 (8)	0.0395 (8)	0.0401 (8)	−0.0048 (7)	−0.0030 (6)	−0.0010 (7)
C8	0.0428 (8)	0.0395 (8)	0.0388 (8)	−0.0031 (7)	−0.0020 (6)	0.0002 (7)
C9	0.0436 (8)	0.0394 (9)	0.0392 (8)	−0.0041 (7)	−0.0042 (6)	0.0001 (7)
C10	0.0528 (10)	0.0419 (10)	0.0519 (10)	0.0004 (8)	−0.0098 (8)	0.0005 (8)
C11	0.0578 (11)	0.0509 (11)	0.0595 (12)	0.0041 (9)	−0.0146 (9)	0.0077 (9)
C12	0.0620 (12)	0.0570 (12)	0.0514 (10)	−0.0013 (9)	−0.0214 (9)	0.0007 (9)

### Geometric parameters (Å, °)

**Table:**

Cl1—C1	1.7408 (18)	C2—H2	0.9300
S1—C7	1.6548 (17)	C3—C4	1.393 (2)
S2—C12	1.693 (2)	C3—H3	0.9300
S2—C9	1.7158 (17)	C4—C5	1.388 (2)
O1—C8	1.229 (2)	C5—C6	1.386 (3)
N1—C7	1.336 (2)	C5—H5	0.9300
N1—C4	1.419 (2)	C6—H6	0.9300
N1—H1N	0.87 (2)	C8—C9	1.463 (2)
N2—C8	1.373 (2)	C9—C10	1.374 (2)
N2—C7	1.402 (2)	C10—C11	1.415 (3)
N2—H2N	0.86 (2)	C10—H10	0.9300
C1—C6	1.372 (3)	C11—C12	1.347 (3)
C1—C2	1.382 (3)	C11—H11	0.9300
C2—C3	1.385 (2)	C12—H12	0.9300
			
C12—S2—C9	91.64 (10)	C1—C6—C5	119.94 (17)
C7—N1—C4	131.12 (15)	C1—C6—H6	120.0
C7—N1—H1N	113.5 (14)	C5—C6—H6	120.0
C4—N1—H1N	115.4 (14)	N1—C7—N2	114.12 (15)
C8—N2—C7	129.51 (16)	N1—C7—S1	128.69 (13)
C8—N2—H2N	113.9 (16)	N2—C7—S1	117.15 (13)
C7—N2—H2N	116.5 (16)	O1—C8—N2	122.64 (16)
C6—C1—C2	121.18 (16)	O1—C8—C9	121.65 (16)
C6—C1—Cl1	119.75 (14)	N2—C8—C9	115.71 (15)
C2—C1—Cl1	119.06 (15)	C10—C9—C8	131.32 (16)
C1—C2—C3	118.96 (17)	C10—C9—S2	111.10 (13)
C1—C2—H2	120.5	C8—C9—S2	117.57 (13)
C3—C2—H2	120.5	C9—C10—C11	112.03 (17)
C2—C3—C4	120.61 (16)	C9—C10—H10	124.0
C2—C3—H3	119.7	C11—C10—H10	124.0
C4—C3—H3	119.7	C12—C11—C10	112.44 (18)
C5—C4—C3	119.35 (16)	C12—C11—H11	123.8
C5—C4—N1	125.26 (16)	C10—C11—H11	123.8
C3—C4—N1	115.33 (15)	C11—C12—S2	112.79 (15)
C6—C5—C4	119.96 (18)	C11—C12—H12	123.6
C6—C5—H5	120.0	S2—C12—H12	123.6
C4—C5—H5	120.0		

### Hydrogen-bond geometry (Å, °)

**Table:**

*D*—H···*A*	*D*—H	H···*A*	*D*···*A*	*D*—H···*A*
N1—H1N···O1	0.87 (2)	1.91 (2)	2.651 (2)	143 (2)
C12—H12···Cl1^i^	0.93	2.69	3.523 (2)	149

Symmetry codes: (i) *x*−5/2, −*y*+1/2, *z*−1/2.
